# B Cell Recognition of the Conserved HIV-1 Co-Receptor Binding Site Is Altered by Endogenous Primate CD4

**DOI:** 10.1371/journal.ppat.1000171

**Published:** 2008-10-03

**Authors:** Mattias N. E. Forsell, Barna Dey, Andreas Mörner, Krisha Svehla, Sijy O'dell, Carl-Magnus Högerkorp, Gerald Voss, Rigmor Thorstensson, George M. Shaw, John R. Mascola, Gunilla B. Karlsson Hedestam, Richard T. Wyatt

**Affiliations:** 1 Vaccine Research Center, National Institutes of Health, Bethesda, Maryland, United States of America; 2 Department of Microbiology, Tumor and Cell Biology, Karolinska Institutet, Stockholm, Sweden; 3 Swedish Institute for Infectious Disease Control, Solna, Sweden; 4 GlaxoSmithKline Biologicals, Rixensart, Belgium; 5 University of Alabama in Birmingham, Birmingham, Alabama, United States of America; The Salk Institute for Biological Studies, United States of America

## Abstract

The surface HIV-1 exterior envelope glycoprotein, gp120, binds to CD4 on the target cell surface to induce the co-receptor binding site on gp120 as the initial step in the entry process. The binding site is comprised of a highly conserved region on the gp120 core, as well as elements of the third variable region (V3). Antibodies against the co-receptor binding site are abundantly elicited during natural infection of humans, but the mechanism of elicitation has remained undefined. In this study, we investigate the requirements for elicitation of co-receptor binding site antibodies by inoculating rabbits, monkeys and human-CD4 transgenic (huCD4) rabbits with envelope glycoprotein (Env) trimers possessing high affinity for primate CD4. A cross-species comparison of the antibody responses showed that similar HIV-1 neutralization breadth was elicited by Env trimers in monkeys relative to wild-type (WT) rabbits. In contrast, antibodies against the co-receptor site on gp120 were elicited only in monkeys and huCD4 rabbits, but not in the WT rabbits. This was supported by the detection of high-titer co-receptor antibodies in all sera from a set derived from human volunteers inoculated with recombinant gp120. These findings strongly suggest that complexes between Env and (high-affinity) primate CD4 formed *in vivo* are responsible for the elicitation of the co-receptor-site-directed antibodies. They also imply that the naïve B cell receptor repertoire does not recognize the gp120 co-receptor site in the absence of CD4 and illustrate that conformational stabilization, imparted by primary receptor interaction, can alter the immunogenicity of a type 1 viral membrane protein.

## Author Summary

A major goal of HIV-1 vaccine research is to design novel candidates capable of neutralizing the vast array of viruses circulating in the human population. One approach is to base the vaccine upon the HIV-1 outer surface envelope glycoproteins to generate antibodies. However, during persistent infection in humans, the HIV-1 envelope glycoproteins have evolved structural features that limit the elicitation of broadly neutralizing antibodies. These immune “decoys” divert the antibody response resulting in virus subpopulations that can escape the host response. A potential means by which the virus elicits these decoy responses comes as a by-product of the entry process. Binding of the HIV-1 envelope glycoproteins to the primary receptor, human CD4, induces the formation of a second co-receptor binding site on the envelope glycoproteins, which then binds to another protein required for viral entry. Antibodies to the co-receptor binding site are generally ineffective at neutralizing HIV-1 patient isolates. Here, we demonstrate the mechanism by which antibodies to the HIV-1 co-receptor binding site are elicited in animals and humans injected with HIV-1 envelope glycoproteins and describe the implications of their formation regarding natural HIV-1 infection and vaccine design.

## Introduction

The human immunodeficiency virus (HIV-1) exterior envelope glycoprotein, gp120, and the transmembrane glycoprotein, gp41, are non-covalently associated to comprise the trimeric, functional viral spike. These glycoproteins mediate entry and represent the sole virally encoded targets for neutralizing antibodies (nAbs) on the surface of the virus. The HIV-1 envelope glycoproteins, and those from related immunodeficiency viruses, are somewhat unusual in that they mediate target-to-membrane fusion by receptor-triggered conformational changes rather than by low pH-mediated fusion events typified by the influenza virus type 1 viral membrane protein, hemagglutinin (HA) [1]. The interaction of gp120 with the primary receptor, CD4, induces formation or exposure of a bridging sheet mini-domain that is, along with elements of the gp120 third variable region (V3), involved with binding to the co-receptor, CCR5 [2],[3],[4].

As was previously shown, antibodies against this induced co-receptor binding site are abundantly generated during natural HIV infection [5] and may be in part elicited due to the unique ability of gp120 to undergo receptor-induced conformations required for the sequential entry process. The co-receptor site antibodies are termed CD4-induced (CD4i) because following CD4 binding to gp120 (which functionally induces the co-receptor binding), these antibodies bind with enhanced affinity to gp120. The prototype for the co-receptor-directed, CD4i antibodies is 17b. However, it is less well appreciated that several full-length gp120 proteins actually are recognized by CD4i antibodies like 17b with high affinity (or avidity) even in the absence of the primary receptor [6]. The co-receptor-directed antibodies do not generally neutralize most circulating isolates [7]. However, these antibodies have attracted considerable interest due to the remarkable post-translational sulfation of a subset of these antibodies that mimics the functionally important sulfation of the CCR5 co-receptor N-terminus and their selective VH gene usage [8],[9]. Viral evasion of the CD4i antibodies likely occurs due to the in vivo selection for viruses that occlude or do not form this highly conserved region until the virus interacts with the primary receptor, CD4 [7],[10]. Once formed, the conserved site interacts with the largely invariant HIV co-receptor, CCR5. In contrast to the ability of affinity-matured CD4i antibodies, which can recognize the co-receptor site in the absence of CD4 with high functional affinity, the requirements for the naïve B cell receptor to recognize the same site is not presently understood and may differ from that of a mature CD4i antibody. Therefore, one aim of this study was to determine if previously described soluble envelope glycoprotein trimeric immunogens [11] might elicit CD4i antibodies in primates that possess a CD4 that is capable of a high-affinity interaction with the viral spike.

As an immunogen, monomeric gp120 does not elicit broadly nAbs [12] and has failed as a vaccine in a large clinical trial [13]. Therefore, much of the field has moved toward the design of soluble trimeric Env immunogens that more closely mimic the functional spike [11],[14],[15],[16],[17],[18]. The gp140 trimers which we have studied are derived from a neutralization resistant primary isolate, YU2, and are stabilized by heterologous trimerization domains (foldon) and somewhat improve the elicitation of neutralizing antibodies when inoculated into small animals possessing CD4 molecules that do not interact with gp120 [19],[20]. However, these stabilized trimeric immunogens have not been extensively tested in primates, which possess CD4 molecules capable of high affinity interaction with HIV-1 Env. Here, we demonstrate directly that the elicitation of CD4i antibodies by Env trimers is dependent upon the in vivo presence of high-affinity CD4 found in primates, but not present in the wild-type (WT) rabbits. We definitively demonstrate that the presence of endogenous primate CD4 is sufficient to generate CD4i antibodies following inoculation of these same trimers into rabbits rendered transgenic for human CD4. Consistent with these data, we also show the presence of co-receptor-directed antibodies in sera from a subset of patients who participated in the non-efficacious VaxGen phase III clinical trial using monomeric gp120 as a candidate vaccine. Our findings provide clear evidence that binding of a type-I viral membrane protein to its primary receptor can lead to its in vivo altered immunogenicity. It also illustrates that, in contrast to antibodies that have the undergone affinity maturation, the naïve B cell receptor repertoire does not recognize the co-receptor binding site with sufficient affinity to elicit antibodies against this region in the absence of primate CD4.

## Results

### Purified soluble Env trimers bind soluble and cell-surface CD4

**Figure 1:**
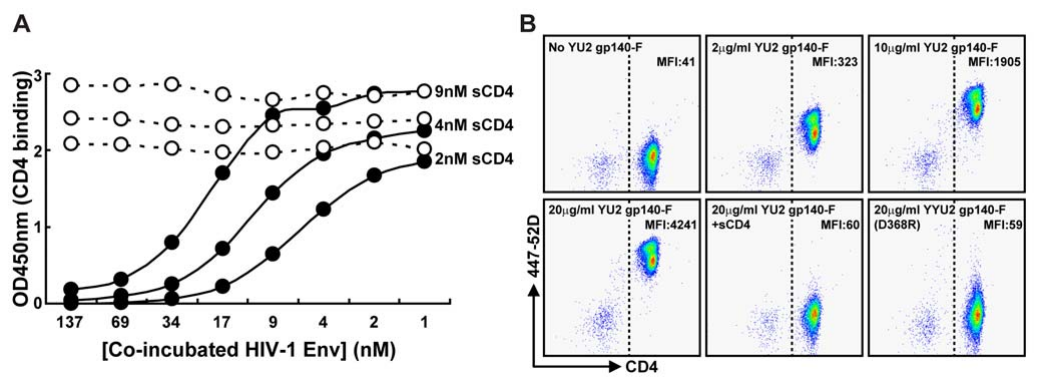
Soluble Env trimers bind to soluble and cell-surface CD4. (A) sCD4 at concentrations of 2, 4 and 9 nM was co-incubated with YU2 gp140-F trimers (filled circles) or CD4-binding-defective gp140 368D/R trimers (open circles) at concentrations shown. Subsequently, non-trimer-bound sCD4 was captured in an ELISA format by the anti-CD4 antibody RPA-T4, competing with HIV-1 Env for CD4 binding. RPA-T4-captured sCD4 was detected with the non-competing anti-CD4 antibody, OKT-4 and plotted as shown. (B) Cynomolgus macaque PBMCs were incubated with 2, 10 or 20 µg/ml of YU2 gp140-F trimers, 20 µg/ml gp140-F trimers in the presence of an excess sCD4 (100 µg/ml) or with 20 µg/ml of gp140-F 368D/R trimers. Cells were stained for CD3, CD4 and CD8 expression and analyzed by flow cytometry. The CD3^+^/CD8^−^ populations are shown (see supplemental FigS2 for gating strategy). The y-axis indicates Env binding, as detected with mAb 447-52D, and the x-axis shows CD4 expression. The CD4^+^ cell population was defined as cells detected to the right of the dotted line. The median fluorescence intensity (MFI) of Env binding to the CD3^+^/CD4^+^/CD8^−^ cell population is shown.

The highly glycosylated gp140-F trimers derived from the primary isolate YU2; previously referred to as YU2gp140(-)/FT [11] were purified from the supernatant of transiently transfected mammalian cells by lentil lectin affinity chromatography followed by chelation chromatography. In most cases, size exclusion chromatography was used to isolate the predominant trimeric peak fraction (Fig. S1). To confirm binding of the trimers to sCD4 independent of avidity effects, a solution-based binding assay was developed. To begin, 1 to 137 nM of the gp140-F molecules were co-incubated with 2, 4 or 9 nM soluble human 4-domain CD4 (sCD4) in solution. Next, non-Env-bound sCD4 was captured by RPA-T4 and detected in an ELISA format to evaluate the relative binding (Fig. 1A). The gp140-F trimers bind to sCD4 in a concentration dependent manner with half-maximal binding at approximately 7, 13 and 26 nM respectively. To confirm the specificity of the binding, we introduced a mutation at position 368 of gp140 such that 368 Asp was changed to Arg. This mutation (368D/R) was previously shown to specifically reduce or abrogate CD4 binding of monomeric gp120 [21] and as expected in this soluble CD4 reporter assay, the gp140 368D/R trimers did not bind sCD4 at any concentration tested. Since *in vivo*, abundant cell-surface CD4 is a potential source for high-affinity binding of Env, we sought to confirm that the YU2 trimers could bind to CD4-positive cells derived from non-human primates before initiating immunogenicity studies. We co-incubated primate peripheral blood mononuclear cells (PBMCs) with 2 µg/ml, 10 µg/ml or 20 µg/ml gp140-F trimers and stained the cells for CD3, CD4, CD8 and a marker for dead cells. Trimer binding to cell-surface-expressed CD4 was detected with the V3-directed antibody 447-52D on live, CD3^+^/CD4^+^/CD8^−^ cells by flow cytometry (Fig. 1B; for staining and gating strategy, see Fig. S2). Similar to the results obtained in the CD4 solution assay, the gp140-F trimers bound to the CD4^+^ T cells in a concentration- dependent manner. Trimer binding to the CD4^+^ cells could be fully abrogated by pre-incubation of 20 µg/ml of the gp140-F molecules with an excess of sCD4. Further, no binding could be detected after incubation of the PBMCs with the gp140 368D/R CD4 binding-defective trimers. Together, the data confirmed that the YU2 gp140-F trimers used in this study bind both soluble, and importantly, cell-surface CD4 in a dose-dependent and specific manner.

### Co-receptor binding site directed antibody recognition of the gp140-F trimers

**Figure 2:**
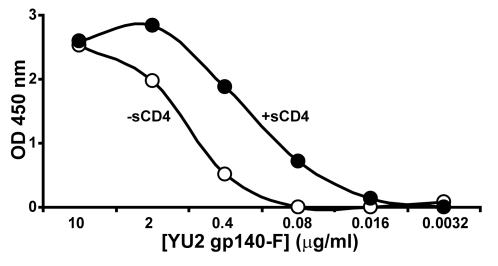
ELISA analysis of the 17b:trimer interaction. The YU2 gp140-F trimers, at concentrations of 3.2 ng/ml to 10 µg/ml, were incubated in the presence (closed circles) or absence (filled circles) of 20 µg/ml sCD4, after which recognition of the trimers by the co-receptor-binding-site-directed antibody, 17b, was analyzed by ELISA. Because of the oligomeric state of the trimers, this assay likely assesses binding avidity, however, the precise stoichiometry of CD4 occupancy of each trimer or the functional ability of each gp120 subunit within the trimer to bind CD4 is not yet known. The addition of sCD4 to the trimers resulted in an approximate 5-fold shift in the levels of ½ maximal binding, from 9 nM, in the absence of CD4, to 2 nM in the presence of CD4.

To confirm that the highly purified trimers used in this study were competent for recognition by 17b, as well as competent for induction of the CD4i epitope by CD4, we performed both ELISA-based and surface plasmon resonance (SPR) binding assays. First, we incubated 3.2 ng/ml to 10 µg/ml of the YU2 gp140-F trimers in solution, without or with an excess of sCD4, after which gp140-F was captured by 17b on a plate (Fig. 2A). Consistent with previous data with monomeric YU2 gp120 [6],[22],[23], the trimers were well recognized by 17b in the absence of CD4. However, under the conditions of this assay, the relative binding increases approximately 2 to 5-fold in the presence of 1 ug/ml or 20 ug/ml sCD4, confirming that sCD4 induces better exposure, by formation or stabilization, of the CD4i site on the gp120 moieties present in the soluble trimeric context.

**Figure 3:**
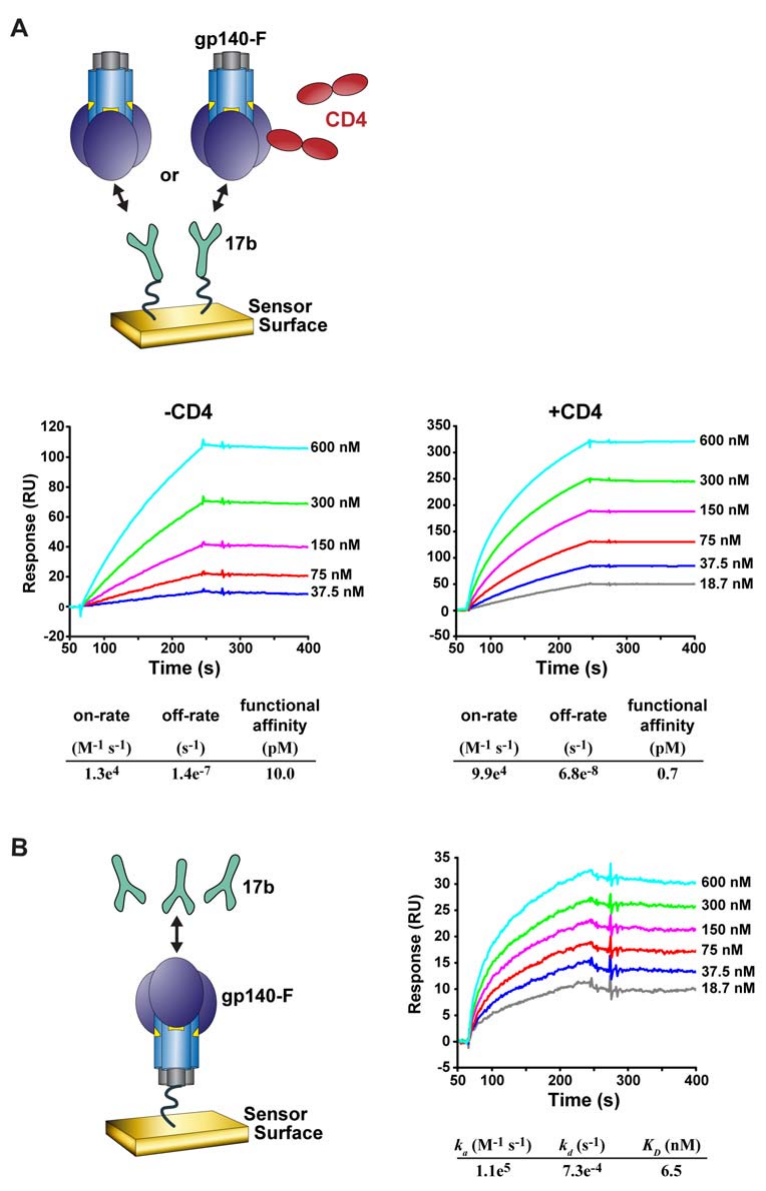
Biacore analysis of 17b:trimer interactions. The recognition of the gp140-F trimers by 17b IgG was assessed by SPR in two formats as shown. (A) 17b IgG was immobilized on the sensor surface and the trimers were then flowed over the Biacore chip either with or without pre-incubation with a 60-fold molar excess of D1D2 CD4. An approximation of the “functional affinity” of each interaction is presented as calculated by 1∶1 Langmuir binding curve fitting. These values likely represent avidity due to the oligomeric state of the gp140-F trimers as the analyte. In this format, the off-rate value was difficult to accurately assess due to the very slow rate of dissociation and may overestimate the functional affinity or avidity. Nevertheless, this is clearly an avid reaction and the on-rates shown here agree with previous reports of the affinity values of monomeric gp120 recognition by monomeric 17b Fab, which is likely the best estimate of affinity [6]. (B) In the other orientation, the trimers were immobilized on the chip surface and 17b was used as the analyte. The affinity was calculated using bivalent curve fitting since the 17b IgG is dimeric. The value derived by this analysis indicates a high-affinity, low nanomolar interaction of the 17b IgG with the trimers in the absence of CD4, as shown.

We next determined the recognition of the trimers by the protypic CD4i antibody 17b by Biacore SPR in two formats (Fig 3). In the first format, the gp140-F trimers were flowed over 17b immobilized on the surface of chip as the analyte. Because the trimers are oligomeric, this binding analysis detects avidity rather than strict affinity. However, this would be the case if the trimers were in solution and recognized by the bivalent BCR in multi-valent array on the surface of a B cell or if the trimers were displayed on the surface of a CD4^+^ lymphocyte. By this means, we determined that the avidity of the trimers for 17b was nanomolar to subnanomolar regardless if the trimers were in complex or not with CD4 (see Fig 3A). To better approximate the actual affinity of interaction, the 17b antibody was flowed over the gp140-F trimers immobilized on the chip and the binding was analyzed by bivalent curve fitting. This analysis also confirmed that recognition of the trimers by 17b in the absence of CD4 was a high-affinity interaction in the low nanomolar range (Fig 3B).

### Env trimer immunogenicity in monkeys and in rabbits

**Figure 4:**
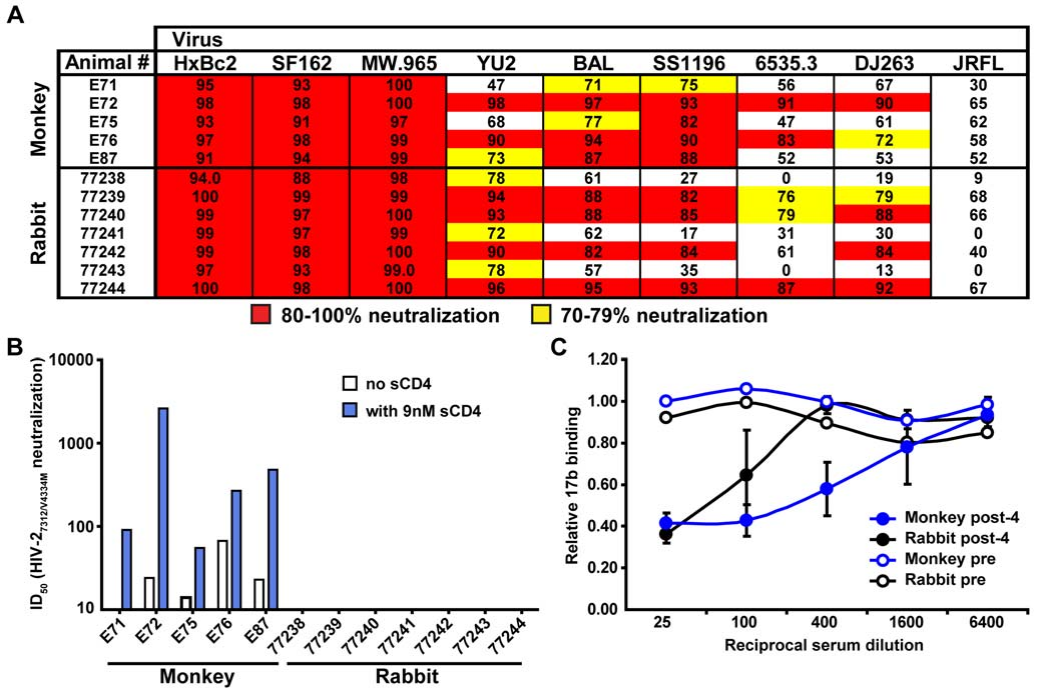
HIV-1 and HIV-2 neutralization. (A) Overall neutralization activity against nine different HIV-1 Env pseudotyped viruses in serum samples from monkeys and rabbits following 4 immunizations with the YU2 gp140-F trimers. Sera were screened at a 1∶5 dilution (except against MW.965, which was done at a 1∶10 dilution of the sera) for neutralization activity. Neutralization between 70 and 79% is indicated in yellow, while neutralization between 80 and 100% is indicated in red. Pre-bleed serum sample effects on viral entry were negligible; BSA adjuvant control animals and MuLV pseudotyped virus were also included as negative controls. (B) Detection of CD4i antibodies in serum samples from immunized cynomolgus macaques and rabbits after 4 immunizations. Serum samples from monkeys and rabbits were titrated for neutralization activity against HIV-2_7312/V434M_. Data are presented as the reciprocal dilution of immune serum resulting in a 50% inhibition of entry (ID_50_) in the absence (white bars) or presence (blue bars) of 9 nM sCD4. (C) A 17b blocking assay was performed using sera elicited from monkeys (blue) and WT rabbits (black). For the assay, the sera were serially diluted in a 96 well format in which the wells were pre-coated with HXBc2 core gp120 glycoproteins. Following washing, biotinylated 17b was added to each well and the ability of the sera to block 17b binding was assessed by ELISA.

To assess if *in vivo* interaction of primate CD4 with HIV-1 Env is a requirement for elicitation of CD4i antibodies, we utilized the previously published observations that WT rabbit CD4 is unable to bind HIV-1 Env [24]. We immunized cynomolgus macaques and rabbits four times with the YU2 gp140-F trimers formulated in the GSK Adjuvant System AS01B and confirmed that the ELISA titers saturated by three inoculations and were roughly equivalent (data not shown). For neutralization, we first analyzed the serum samples from individual animals for their ability to inhibit viral entry against a panel of selected HIV-1 isolates. The rationale for this analysis was to assess the over-all neutralization capacity of responses elicited in monkeys versus the rabbits against HIV-1 to make a comparison of other responses more meaningful (i.e. CD4i-directed HIV-2 neutralizing antibodies, see below). The sera were analyzed at a 1 to 5 dilution against a panel of nine HIV-1 Env pseudotyped viruses in a standardized neutralization assay using TZM-bl target cells [25],[26] (Fig. 4A). Sera derived from animals of both species potently neutralized the three sensitive viruses, the lab-adapted HxBc2 (clade B), SF162 (clade B) and MW.965 (clade C) with values between 90 and 100%. Overall, the potency and breadth of neutralization for this panel of viruses were very similar between the monkey and rabbit sera. Subtle cross-species differences in neutralization were observed, but these were not statistically significant. For example, sera from the immunized rabbits tended to display more consistent animal-to-animal neutralization capacity against the homologous YU2 strain. In contrast, the sera derived from the monkeys displayed a trend of greater potency against BaL and the tier 2 isolate SS1196 (clade B). Sera derived from both species of animals inoculated with the YU2 trimers sporadically neutralized the DJ293 isolate (clade A), but poorly neutralized JRFL (clade B), as well as TRO1.1 (clade B; not shown, done only with monkey sera), demarking the limits of the neutralization activity elicited by the current immunogen design. Perhaps the subtle differences observed in the neutralization potency against some of the isolates between sera derived from the monkeys versus the rabbits is due to slight differentials in the elicited antibody repertoires, however, in general, the data highlights the overall similarities in the elicited neutralization capacity.

To detect if there is a species-difference in specific antibody elicitation against the co-receptor site of gp140-F trimers, we analyzed the sera in the same assay format as above but against virus pseudotyped with Env from an HIV-2 isolate, 7312 (containing a V434M amino acid change). While this virus is relatively insensitive to antibodies raised against HIV-1 Env it becomes highly sensitive to anti-HIV-1 CD4i-antibodies in the presence of sub-inhibitory concentrations of sCD4, facilitating the specific detection of such antibodies [5]. Consistent with data from HIV-1 infected individuals [5],[27] and gp140-inoculated humans (GMS, unpublished observations), CD4i-antibodies detected by this assay were abundant in sera from all five monkeys (ID_50_ titers: 55, 91, 268, 479 and 2618; Fig 4B). We could detect low-level cross-neutralization of HIV-2_7312/V434M_ in sera from four of the five monkeys, consistent with what had been observed previously from some HIV-1 infected humans [5]. Following these results, we analyzed three cynomolgus macaques that had been inoculated with the YU2 gp140-F trimers in Ribi adjuvant 2 times and at a similar dose, and detected CD4i antibodies in these animals with ID 50 values of 21, 27 and 198 (Fig S3). The lower levels of CD4i antibodies relative to those elicited by trimers formulated in AS01B, correlated with similarly lower potency of neutralization against the HIV-1 isolate, MN (Fig S3). We also detected CD4i antibodies in 5 out of 5 cynomolgus monkeys primed with Semliki Forest virus (SFV) particles expressing the YU2 gp140-F trimers and boosted with trimeric protein with ID_50_ values of 57, 1619, 44 and 335 and in 3 out of 3 baboons immunized with YU2 gp140 molecules rendered trimeric with a modified GCN4 motif [16] in Ribi adjuvant (data not shown). Taken together, these data clearly demonstrate that elicitation of CD4i antibodies by Env trimers in non-human primates is a highly reproducible and commonly elicited response and can occur at low levels when Env is expressed from a viral vector (SFV; not shown).

In stark contrast, CD4i-antibodies could not be detected in the serum from any of the WT rabbits (Fig 4B), suggesting that the elicitation of CD4i-antibodies is dependent on the *in-vivo* presence of CD4 with affinity for the YU2 trimers in the non-human primates. Alternatively, it might be that rabbits lack B cell receptors (BCR) in their naïve repertoire with the ability to recognize the HIV-1 co-receptor binding site while the monkeys possess such a capacity.

While the HIV-2 assay detects antibodies specific for the co-receptor binding site, we wanted to confirm these results by performing an ELISA based assay where serum from immunized animals were tested for their ability to compete with a biotinylated 17b antibody for binding to gp120. It is known that antibodies not directly directed against the co-receptor site are capable of competing with 17b for binding to gp120 [28]. To minimize such indirect effects, we analyzed the ability of sera to compete for biotinylated 17b binding to an HXBc2 gp120 core protein capable of binding 17b with high affinity (Dey et al, manuscript in preparation). In this assay format serum samples from monkeys were able to inhibit 17b binding at an approximately 10-fold higher dilution than that of serum samples from the rabbits (Fig 4C). The weak, but detectable, level of antibodies capable of competing with 17b in serum from rabbit serum may be due to antibodies recognizing the co-receptor binding elements in the pre-CD4 induced state or other antibodies that can inhibit 17b binding, such as CD4 binding site antibodies [28].

Direct interaction of the trimers with primate CD4 might be expected to expose as well V3, the other element of gp120 involved in co-receptor interaction [4]. To address this issue, we performed a binding assay to determine the proportion of V3-specific antibodies compared to antibodies against intact gp120 in sera derived from the 3 types of test animals. We observed a slight 1.6-fold average higher proportion of antibodies against V3 in serum samples from primates than in WT rabbits that was not statistically significant (Fig S3). The huCD4 rabbits, although possessing lower gp120 and V3 binding titers compared to WT animals, also displayed a slightly greater proportion of V3-specific antibodies. The lack of a significant increase in V3-directed antibodies may be due to maximal exposure of V3 on gp140 as we could see no enhancement of binding of a V3-specific antibody to the trimers following addition of CD4 (not shown) consistent with a previous study [28].

### Immunization of human-CD4-transgenic rabbits with trimers

**Figure 5:**
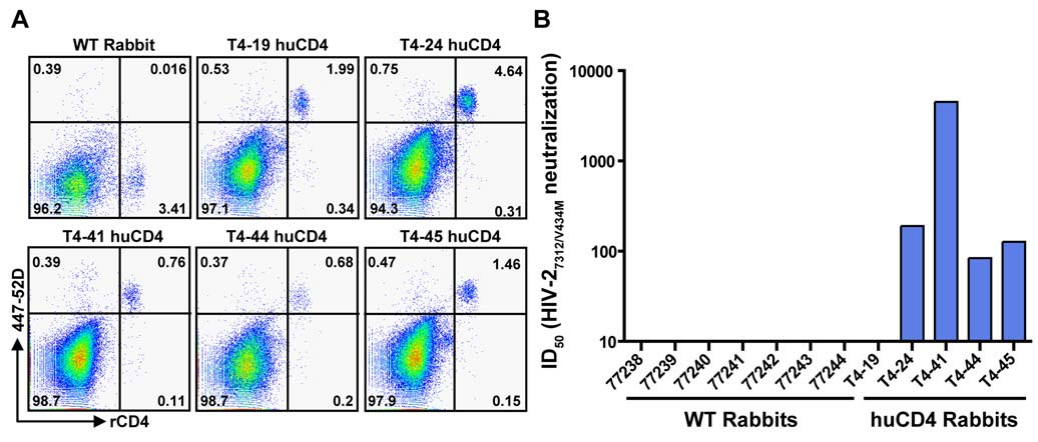
Characterization of huCD4 rabbit PBMCs and HIV-2 neutralization. (A) CD4 expression and YU2 gp140-F trimer binding to PBMCs from WT and huCD4 rabbits (T4-19, T4-24, T4-41, T4-44 and T4-45) was analyzed by flow cytometry. PBMCs were incubated with 20 µg/ml of the gp140-F trimers and stained with an anti-rabbit CD4 antibody (x-axis) followed by detection of trimer binding with the mAb 447-52D (y-axis) (see supplemental Fig S5 for gating strategy). rCD4^+^/Env^−^ cells are located in the lower right quadrant while rCD4^+^/Env^+^ cells are located in the upper right quadrant. The percentage of cells detected in each quadrant is indicated. (B) ID_50_ HIV-2_7312/V434M_ neutralization in the presence of 9 nM sCD4 by serum samples from WT and huCD4 rabbits (blue) after 3 immunizations with the gp140-F trimers.

To address if the presence of primate CD4 was required for the elicitation of CD4i antibodies from the gp140-F trimeric immunogens, we used rabbits previously engineered to be transgenic for human CD4 (huCD4) [24]. These rabbits were generated from the New Zealand White (NZW) background, and are relatively similar in their genetic background to the out-bred NZW WT rabbits used for the initial immunogenicty analysis above. The huCD4 transgenic rabbits allowed us to perform controlled immunogenicity experiments to determine if the *in vivo* presence of primate CD4 allows for BCR recognition of the YU2 gp140-F co-receptor site and subsequent elicitation of CD4-induced antibodies in rabbits. Before initiating the immunogenicity experiment, we confirmed that the huCD4 transgenic animals, ranging from 2 to 5 years of age, still expressed human CD4 on their PBMCs as follows. Incubation of 20 µg/ml gp140-F trimers with PBMCs from WT and huCD4 rabbits and analysis by flow cytometry using species-specific cellular makers (for staining and gating strategy, see Fig. S5) confirmed that only PBMCs from the transgenic animals can bind the gp140-F, and that binding occurred only on cells co-expressing human and rabbit CD4 (rCD4; Fig. 5A). These results are consistent with the initial design of the transgenic rabbits to restrict expression of huCD4 only to cells also co-expressing rCD4 by use of a cell-type-specific promoter [24].

Five huCD4 rabbits were inoculated with the YU2 gp140-F trimers formulated in AS01B adjuvant by an identical regimen as the WT rabbits and monitored for the appearance of CD4-induced antibodies by the *in vitro* HIV-2 assay. CD4-induced antibodies could be detected in the sera from four out of five huCD4 rabbits after three immunizations with gp140-F trimers (Fig 5B). The ID_50_ titers detected were 84, 127, 190 and 4507, which is comparable with the levels detected in serum samples derived from immunized monkeys. These data demonstrate that rabbits have the capacity to induce antibodies against the HIV-1 co-receptor site, but that the *in vivo* presence of primate CD4 is required for the elicitation of these antibodies. This most likely occurs by a direct interaction with primate CD4 and induction of the co-receptor binding site, consistent with a recent study that detects CD4i antibodies following inoculation of monkeys with a CD4-gp120 fusion protein [29]. We also monitored for elicitation of neutralizing antibodies against HIV-1 pseudotyped virus in serum samples of these huCD4 rabbits and observed that the titers were not as consistent and potent for as for the WT rabbits (data not shown). These results might be due to the relatively advanced age of the huCD4 rabbits or as a consequence of unanticipated immune-related effects of the huCD4 transgene. However, as a slightly diminished immune response in these animals would only bias the results against the elicitation of CD4i antibodies, this remains a stringent model to assess the dependence upon primate CD4 for elicitation of these antibodies.

### The 17b antibody can recognize cell-surface, CD4-bound soluble Env trimers

**Figure 6:**
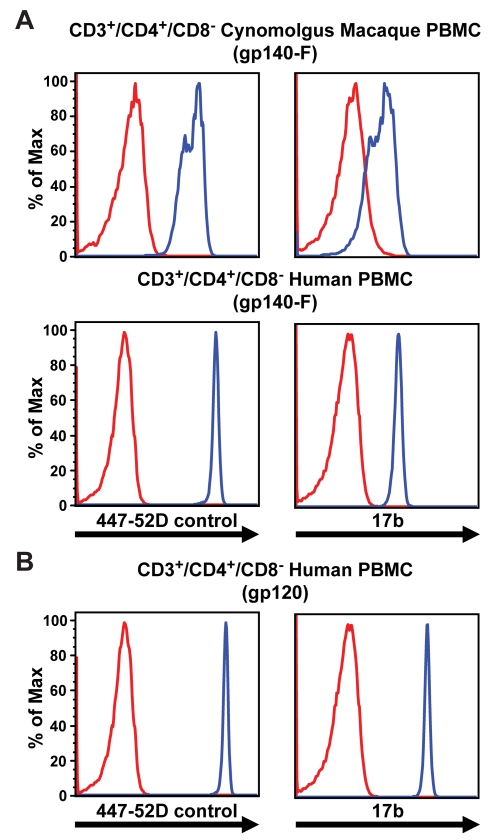
Accessibility of the HIV-1 co-receptor site after CD4 binding. (A). PBMCs from cynomolgus macaques and humans were co-incubated with 20 µg/ml YU2 trimers or (B) with 5 µg/ml YU2gp120 (only human PBMCs). CD4 specific trimer binding (blue) to CD3^+^/CD4^+^/CD8^−^ cells was detected with either the V3-directed mAb, 447-52D (left panels), or with the co-receptor site-directed mAb, 17b (right panels), by flow cytometry analysis. Negative control staining (red, both panels) shows the fluorescence signal obtained in the absence of 447-52D or 17b.

The elicitation of CD4i antibodies in monkeys and huCD4 rabbits after immunization with YU2 trimers suggests that the co-receptor site of gp120 is accessible for BCR recognition: likely on the surface of CD4^+^ PBMCs. Therefore, we investigated if the prototypic, co-receptor-site-directed mAb, 17b, could bind gp140-F trimers once they were bound cell-surface CD4. We sought to confirm that there was adequate accessibility of the CD4-induced co-receptor binding site on the trimers, once they were removed from the context of the virus. In the viral spike context, the induced co-receptor site is apparently not accessible to most CD4-induced antibodies due to steric constraints. To approximate the *in vivo* scenario in which trimers formulated in adjuvant would likely drain to proximal lymph nodes to encounter abundant CD4^+^ cells, we incubated cynomolgus macaques PBMCs with 20 µg/ml of the gp140-F trimers and detected CD4-specific binding to live CD3^+^/CD4^+^/CD8^−^ cells by the V3-directed antibody 447-52D or 17b using flow cytometry (Fig. 6A). Binding of the gp140-F trimers could be detected with both antibodies. Recognition of the trimers by 447-52D verifies that the YU2 gp140-F molecules bind to the CD4^+^ cells, while cell-surface recognition of the trimers by 17b confirms that the co-receptor site is accessible after trimer binding to membrane-bound CD4. Similar results were obtained when the cell-surface binding assay was repeated using human PBMC targets as shown in Fig 4B. Monomeric gp120 bound to the human CD4^+^ cells was used as a control and displayed the 17b epitope at levels higher than that of the gp140-F trimers (Fig 6B).

Sera from humans immunized with gp120 possess CD4i antibodies. Following the observation that the gp120 monomers bound to cell-surface CD4 displayed the CD4i epitope, we obtained serum samples from the VaxGen Inc phase III clinical trial now licensed to the Global Solutions for Infectious Diseases. Twenty randomly selected sera from trial volunteers that had been inoculated four times with recombinant gp120 (MN/GNE8 mixture) formulated with alum were assessed for gp120 binding antibodies by ELISA. All sera exhibited detectable binding titers to the unmatched YU2 gp120 ranging from 5000 to 100,000 endpoint titers (not shown). We assessed the ability of the sera to inhibit the entry of MN and, in the presence of CD4, the HIV-2 virus 7312. As shown in Table 1, all sera neutralized not only the homologous virus, MN, but displayed detectable, relatively high-titer, cross-neutralizing, co-receptor-directed antibodies against the CD4-triggered HIV-2 isolate.

**Table 1:** Neutralization values present in sera elicited by gp120 inoculated into humans

Serum sample	VIRUS			
	HIV-2 7312/V4334M+9nM sCD4		HIV-1 MN	
	ID_50_	ID_80_	ID_50_	ID_80_
**001**	4,178	737	24,410	1,925
**002**	74,789	10,124	141,655	12,343
**003**	8,890	1,145	11,180	1,051
**004**	2,452	365	1,181	216
**005**	15,110	2,279	9,134	1,912
**006**	1,286	309	1,116	91
**007**	1,664	406	8,496	577
**008**	2,002	391	2,340	378
**009**	2,974	642	46,508	1,485
**010**	3,226	666	20,787	1,619
**011**	1,229	226	8,962	369
**012**	630	94	1,532	14
**013**	3,601	548	20,572	1,143
**014**	1,864	339	21,759	1,313
**015**	292	80	1,351	282
**016**	5,415	1,162	6,687	967
**017**	1,904	376	31,204	2,083
**018**	3,571	661	2,579	383
**019**	430	115	302	87
**020**	762	132	6,406	644

## Discussion

**Figure 7:**
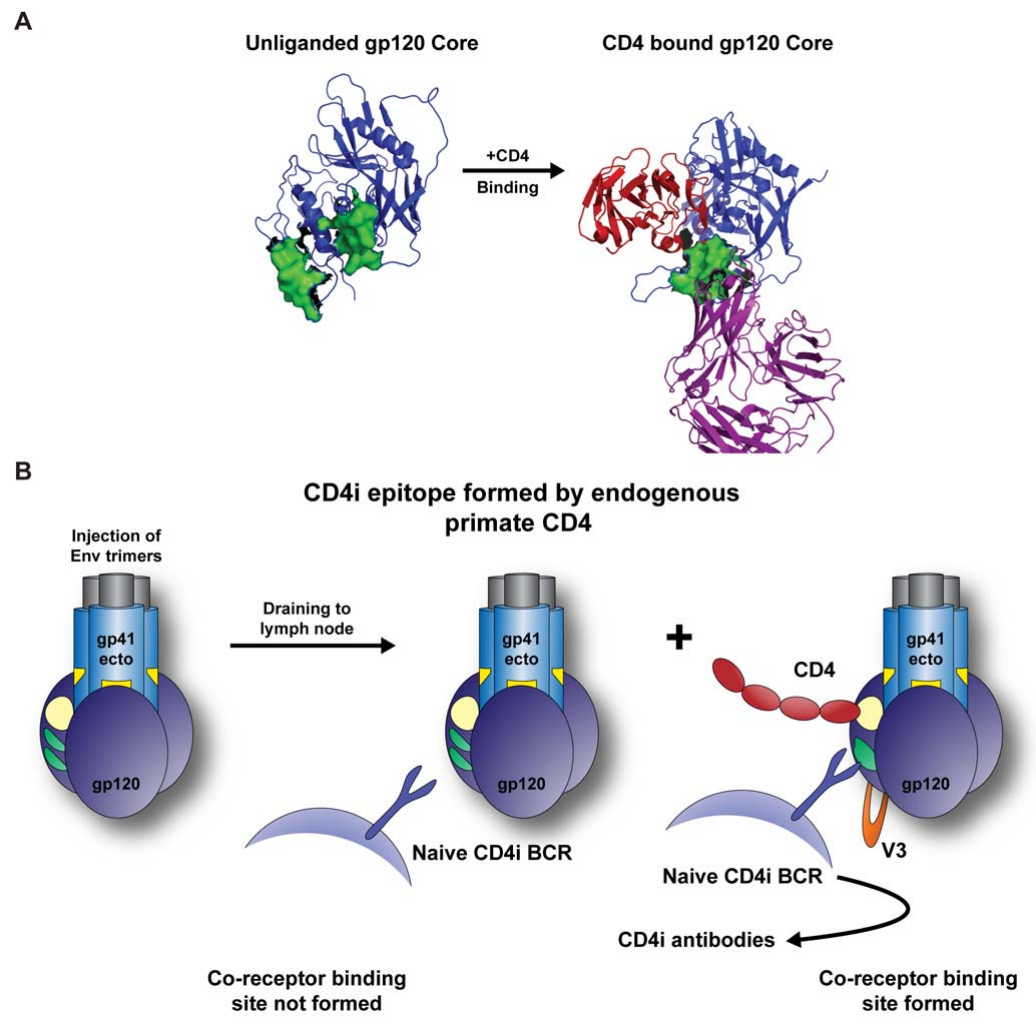
Schematic representations of the pre-CD4 and post-CD4 conformations of Env. (A) Based upon the structures of the unliganded SIV core gp120 [40] and the CD4-bound HIV-1 core [41], the movements of the co-receptor site bridging sheet beta-strands are highlighted in green. Note that 17b binds to the unliganded HIV-1 core with low affinity (∼1 uM) consistent with the “split orientation” of the bridging sheet revealed by the unliganded SIV core structure. 17b can also recognize full-length gp120 with high affinity [6] and the gp140 trimers as demonstrated in this study (see Figs 2 and 3). These observations suggest that the bridging sheet may not be in exactly the same conformation in the full-length protein context as it is in the original gp120 core protein structures with and without CD4. (B) A model of how the co-receptor region (green) is recognized by the naïve B cell receptor repertoire. In the left and middle panel, the co-receptor binding site is not formed. Following binding to high-affinity primate CD4 (likely on the surface of CD4^+^ cells), the bridging sheet is formed and locked into a single, CD4-dependent conformation, which then allows elicitation of co-receptor-site-directed, CD4i antibodies (right panel). The somatically mutated and affinity-matured 17b antibody can recognize either conformation, presumably inducing a proper fit of the unliganded bridging sheet conformation.

In this study, we demonstrate that the elicitation of co-receptor site directed antibodies by the YU2 gp140-F trimers requires the presence of primate CD4. We show that the relatively homogenous, soluble, stable YU2 trimers bind to human CD4 with high affinity in a solution-based assay that, by design, should be independent of oligomeric influences on functional affinity by avidity-dependent interactions. Analysis of the interaction between the prototypic co-receptor antibody, 17b, and the trimers demonstrated that high-affinity and high-avidity binding is detectable even in the absence of CD4. Binding of the trimers to primate cell-surface CD4, but not to cell-surface rabbit CD4 was also shown. WT rabbits and monkeys inoculated with the CD4-binding YU2 Env trimers formulated in the same adjuvant system elicited an overall similar pattern of HIV-1 *in vitro* neutralization against the viruses tested. However, cross neutralization of HIV-2 in presence of sCD4, an assay that is diagnostic for the detection of CD4i antibodies, was observed initially in sera derived from monkeys inoculated with the YU2 trimers but not in WT rabbits. Taken together, these data strongly suggest that Env-CD4 complexes generated *in vivo* upon inoculation are the source for elicitation of the CD4-induced antibodies following vaccination. This observation was confirmed by the inoculation of Env trimers into rabbits transgenic for human CD4 and the detection of CD4i antibodies in the sera of these animals, in contrast to WT rabbits, revealing conclusively the mechanism of their generation (see schematic Fig 7). Consistent with this observation, high levels of co-receptor-directed, HIV-2 (+CD4) cross neutralizing antibodies were detected in 20 of 20 human serum samples from the VaxGen phase III clinical trial using monomeric gp120 [13], suggesting that during natural infection shed, soluble gp120 can elicit these antibodies [30].

The induction of co-receptor site directed antibodies in non-human primates and humans is consistent with previous reports that detected the presence of CD4i, co-receptor directed antibodies in gp140-immunized humans (GMS, unpublished observations), as well as in naturally infected humans [5],[31] and following SHIV challenge of naïve non-human primates [29]. However, the mechanistic basis for the elicitation of CD4i antibodies was not previously addressed in a direct manner. In this study, we present a controlled experiment, which demonstrates that the elicitation of the co-receptor binding site antibodies by Env alone requires the presence of, and likely direct interaction with, primate CD4. This requirement has not previously been defined, despite numerous Env trimer immunogenicity experiments performed to date in both monkeys and non-primate species [17],[18],[20],[32],[33]. This is in part, because the HIV-2-based neutralization assay diagnostic for CD4i antibodies was a relatively recent development and is more definitive for neutralizing capacity directed at the co-receptor binding site then are binding assays employed by us and others previously [23],[34],[35]. Elicitation of CD4i antibodies in primates by Env trimers also nicely illustrates another potential mechanism of immune escape by HIV-1. Not only does Env binding to CD4 obscure a conserved surface that, if it was highly immunogenic, might elicit antibodies capable neutralizing a broad array of isolates (essentially antibodies mimicking the soluble primary receptor), but the binding event induces a second conserved and apparently immunogenic region: the co-receptor binding site. The CD4i antibodies directed against this region are not generally able to neutralize primary isolates in vitro [7]. This is likely due to a commonly elicited selection pressure that renders the co-receptor binding inaccessible to most antibodies of this type [10]. The inability of the CD4i antibodies to control HIV-1 infection is supported by data from a recent study where elicitation of CD4i antibodies can be detected prior to the detection of autologous virus neutralization capacity in sera derived from HIV-1 infected patients [27], as well as the data here, which indicates that they were elicited in the phase III Vaxgen clinical trial where no protection was observed. However, a recent study in non-human primates suggests that the presence of CD4i antibodies (as determined *in vitro* by the same HIV-2 detection assay as used here) is associated with more rapid viral clearance following SHIV162P3 challenge [29], illuminating that this is an area worthy of further investigation.

In the present study, the most likely *in vivo* source for presentation of the CD4i region to the humoral immune system is by direct interaction of the trimers with cell-surface CD4 displayed on CD4-expressing T cells or other CD4-positive cells of the hematopoetic lineage. It is also possible that low levels of CD4 are shed from CD4^+^ cells into interstitial spaces and soluble complexes are formed. Detection of low levels of sCD4 has been previously reported in humans [36],[37], although in the assay used here we could not detect soluble CD4 in the sera of animals examined. It is also possible that trimer binding to cell-surface CD4 induces shedding of complexes, but we could find no such evidence for soluble Env-CD4 complexes in the sera.

The implications of inducing the co-receptor binding site has been discussed extensively at the level of entry, but less so at the level of antibody elicitation. That the CD4i antibodies are not elicited by trimers in the absence of CD4, even though the gp140-F molecules are well recognized by 17b, and that CD4 induction of the epitope in the trimer context is not a requirement for 17b binding, may seem to be a bit of a paradox. However, we interpret these data to indicate that the conformational fixation imparted by CD4 binding to gp120 is a critical requirement for the naïve, germline B cell repertoire to efficiently recognize the site as opposed to the affinity-matured 17b antibody, which can likely induce the fit of its epitope in the absence of CD4 (see Schematic Fig 7). This is more likely to be an affinity limitation of the non-affinity matured BCR repertoire, although it is possible that it is somehow related to binding limitations of Ig molecules on the surface of B cells and epitope accessibility issues.

Another possible interpretation of the data is that elements of the pre-CD4 co-receptor binding site are immunogenic, but do not elicit antibodies that cross react efficiently with the fully formed site induced by CD4. However, the 17b blocking assay, using a form of the gp120 core with the potential to be recognized by any antibodies to the co-receptor site indicated that the pre-CD4 state of the trimers did not elicit many antibodies directed toward this region compared to those elicited in the presence of primate CD4 (Fig 4C). Also, we cannot rule out that array of gp140 or gp120 on the surface of CD4+ cells might also enhance the elicitation of CD4i antibodies in a manner independent of conformational fixation. However, the study by DeVico *et al* [29] clearly demonstrates that covalent gp120-CD4 complexes incapable of binding cell-surface CD4 efficiently elicit CD4i antibodies, so CD4-dependent cell-surface array cannot be the only explanation for their elicitation in the presences of primate CD4.

The implications of the data presented here are also an important consideration for vaccine candidates designed to elicit neutralizing antibodies against the conserved gp120 CD4 binding site. The Env CD4bs likely remains fully accessible in animals without human or primate CD4, however the elicitation of the CD4i antibodies in animals with primate CD4 indicates that this is likely not the case in species harboring CD4 molecules with a high affinity to Env. These results suggest that a fraction of the population of a CD4-binding-competent immunogen will interact with primate CD4 and thereby occlude the CD4 binding region on this protein subset. It is possible that the subtle differences detected in the neutralization profile between WT rabbits and monkeys occur as a result of such an interaction, partially altering the spectrum of antibodies that are elicited. However, the fractional component of the inoculate which binds to CD4 as yet remains to be determined, and may not be absolute as the overall HIV-1 neutralization profile elicited by the trimers used in this study was similar between the rabbits and the non-human primates.

It was also previously shown that HIV Env-CD4 interaction resulted in altered CD4^+^ T cell function *in vitro* [38] and it was suggested that elimination of Env interaction with CD4 in the context of vaccination might be beneficial to better elicit functional T cell help and more potent neutralizing antibody responses. From that study and the data presented here it will be interesting to assess if Env variants that do not bind CD4, but still retain the ability to bind CD4 binding site antibodies might make better immunogens than do unmodified YU2 gp140-F proteins. Alternatively, redirecting the immunogen more efficiently to B cell and antigen presenting cells might also overcome any potential detrimental effects of Env-based immunogens interacting with primate CD4. Follow up immunogen trimer design, characterization and immunogenicity studies are warranted to clarify these issues further in the near future.

## Materials and Methods

### Protein production and purification

Proteins were produced by transient transfection of adherent 293F cells or 293Freestyle suspension cells. The highly glycosylated and His-tag containing YU2gp140-F trimers were captured and purified from the serum-free media in a three-step process. First, the protein was captured via glycans over with lentil-lectin affinity chromatography (GE Healthcare, Uppsala, Sweden). After extensive washing with PBS the protein was eluted and captured in the second step via the His-tag by nickel-chelation chromatography. (GE Healthcare) then washed and eluted with a 300 mM Imidazole containing PBS buffer. In some cases the YU2gp140-F trimers were separated from lower molecular weight forms by the third step of gel filtration chromatography using a superdex200 26/60 prep grade column by the ÄKTA Fast protein liquid chromatography system (GE Healthcare). In contrast, the YU2gp120 and HXBc2 gp120 core proteins were purified by capturing the molecules on an IgG17b affinity column. After extensive washing with PBS, the proteins were eluted from the column with 100 mM glycine/Tris HCl/150 mM NaCl. pH 2.8 and immediately neutralized with Tris base, pH 8.5.

### CD4 solution competition assay

Env protein was co-incubated at concentrations of 0.4 to 46 nM in PBS with 2, 4 or 9 nM sCD4 at room temperature for 1 h, allowing for CD4-Env trimer complexes to form. Non-Env bound sCD4 was captured on a plate pre-adsorbed with 200 ng/well of the anti-CD4 antibody RPA-T4 (Ebioscience, San Diego, CA). RPA-T4 binds to domain 1 of CD4 and competes with HIV-1 gp120 for binding. RPA-T4 bound sCD4 was probed with a biotin-conjugated, non-competitive anti-CD4 antibody, OKT-4 (Ebioscience). Horseradish peroxidase (HRP) conjugated streptavidin (Sigma) followed by the colorimetric peroxide enzyme immunoassay substrate (3,3′,5,5′-tetramethylbenzidine; Bio-Rad) was added to induce a colorimetric change and the reaction was stopped by adding 1 M H_2_SO_4_. OD was read at 450 nm.

### Enzyme-linked immunosorbent assay (ELISA)

High-protein-binding MaxiSorp plates (Nunc) plates were coated with 200 ng/well of mAb 17b in 100 µl of PBS at 4°C overnight after which the wells were blocked for 2 h at room temperature (RT) with PBS-2% fat-free milk. The gp140-F trimers, at concentrations between 3.2 ng/ml to 10 µg/ml, were pre-incubated with 20 µg/ml sCD4 for 1 h at RT and then added to the wells. The wells were then probed for 17b bound gp140-F trimer with rabbit anti-gp140-F polyclonal sera. Addition of HRP conjugated anti-rabbit-Ig (Fc region) (Jackson Laboratories, Bar Harbor, MN) followed by the colorimetric peroxide enzyme immunoassay substrate (3,3′,5,5′-tetramethylbenzidine; Bio-Rad) was used to induce a colorimetric change and the reaction was stopped by adding 1 M H_2_SO_4_. OD was read at 450 nm. The 17b binding competition assay was performed by coating the ELISA plate with 200 ng/well of lectin from *Galanthus nivalis* (Sigma), followed by 200 ng/well of HXBc2 core protein. After blocking with 2% fat-free milk, serum was incubated for 45 minutes at dilutions between 25 and 6400 in a total volume of 100 ul after which 25 ul of biotin conjugated 17b antibody was added to a final dilution of 2500 and incubated for an additional 45 minutes. The plate was probed with HRP conjugated streptavidin and developed as above.

### SPR kinetic analysis

To determine the kinetic constants of YU2gp140-F interaction with 17b IgG, we performed binding analysis were in two different formats on a Biacore3000 surface plasmon resonance spectrometer. In one format (Fig 3A and B), YU2gp140-F (without or with pre-binding to 20-fold molar excess of D1D2 sCD4 for 1 h) was passed over the 17b IgG surface. Because of the potential oligomeric interaction of the trimers with 17b IgG presented on the surface of the chip, this analysis likely measured avidity rather than simple affinity. However, since the curves approximated single binding kinetics and we did not know how many monomeric subunits within the trimers were capable of 17b interaction, curve fitting was done assuming a 1∶1 interaction. In the reverse format (Fig. 3C), the 17b IgG was passed over YU2gp140-F surface and functional (or apparent) affinity was calculated using the bivalent analyte program (Biacore) that derived affinity from the potential bivalent interaction of the 17b with trimers immobilized on the chip surface.

To prepare binding surfaces, ligands (7 µg/ml in 10 mM NaOAc, pH 5.5 buffer) were immobilized on CM5 chip by the amine coupling method following manufacturer's protocol. One flow cell receiving only NaOAc buffer was used as reference control for correction of background binding. For binding experiments, analytes were serially diluted at concentrations ranging from 4.6 nM to 600 nM in the HEPES-EP reaction buffer. To determine the rate of association, each analyte was allowed to flow over the activated surfaces at a rate of 30 µl/min for 3 minutes. Dissociation was determined by washing off bound analyte for the next 5 min. Likely due to avidity of the oligomeric analytes, especially in Fig 3A, the rate of dissociation was difficult to determine and likely represents and over estimate of the actual 1∶1 binding kinetics. The surface was regenerated by removing any unbound analyte with two injections (60 sec each) of 10 mM Glycine, pH 3.0. All procedures were performed at RT.

### Flow cytometry

Monkey, human and rabbit PBMCs were analyzed by flow cytometry using a modified LSR I system (BD Biosciences). Data analysis was performed using FlowJo software (Tree Star, San Carlos, CA). Staining and gating strategies to detect YU2 trimer binding to live, CD3^+^/CD4^+^/CD8^−^ cells from primates is described in Fig. S2. Staining and gating strategy for trimer binding to rabbit cells is described in Fig. S4. The mAb 447-52D was a kind gift from Susan Zolla-Pazner (New York University School of Medicine).

### Animals

Five female cynomolgus macaques (*Macaca fascicularis*) of Chinese origin, 5–6 years old, were housed in the Astrid Fagraeus laboratory at the Swedish Institute for Infectious Disease Control. Housing and care procedures were in compliance with the provisions and general guidelines of the Swedish Animal Welfare Agency. The animals were housed in pairs in 4 m^3^ cages, enriched to give them possibility to express their physiological and behavioural needs. They were habituated to the housing conditions for more than 6 weeks before the start of the experiment, and subjected to positive reinforcement training in order to reduce the stress associated with experimental procedures. All immunizations and blood sampling were performed under sedation with ketamine 10 mg/kg intramuscularly (i.m.). (Ketaminol 100 mg/ml, Intervet, Sweden) The macaques were weighed and examined for swelling of lymphnodes and spleen at each immunization or sampling occasion. Before entering the study, all monkeys were confirmed negative for simian immunodeficiency virus (SIV), simian T-cell lymphotropic virus and simian retrovirus type D. Female New Zealand White Rabbits and male huCD4 New Zealand White transgenic rabbits were housed at BioCon, Inc (Rockville, MD) or at an animal facility at the National Institutes of Health according to current regulations. Cynomolgus macaques were injected once with 200 ug followed by three injections with 100 µg YU2gp140-F trimer. Rabbits were injected four times with 50 ug YU2 trimer. All proteins were formulated in the GSK-AS01B adjuvant system (GlaxoSmithKline, Rixensart, Belgium) prior to injection unless otherwise stated and all injections were administered i.m. at an interval of one month. Sera were collected before the first injection as well as two weeks after each injection. All procedures were approved by the Local Ethical Committee on Animal Experiments.

### Neutralization Assays

Analysis for HIV-1 and HIV-2 neutralization in serum samples were performed as previously described [25],[26]. Briefly, Env pseudoviruses were prepared by co-transfecting 293T cells with an Env expression plasmid containing a full gp160 env gene and an *env*-deficient HIV-1 backbone vector (pSG3 Env). For screening, a single dilution of sera or plasma was used and the percent neutralization was calculated compared to controls with no sera added. To determine the dilution of the sera that resulted in a 50% reduction in RLU against selected viruses, serial dilution assays were performed and the neutralization dose-response curves were fit by non-linear regression using a 4-paremeter hill slope equation programmed into JMP statistical software (JMP 5.1, SAS Institute Inc., Cary, NC). The results are reported as the serum neutralization ID_50_, which is the reciprocal value of the serum dilution resulting in a 50% reduction in viral entry. Dana Gabuzda (Dana Farber Cancer Institute) provided the Env plasmid for YU2. Env plasmids for SF162 and JRFL were provided by Leonidas Stamatatos (Seattle Biomedical Research Institute) and James Binley (Torrey Pines Institute), respectively. The Clade A Env-pseudovirus DJ263.8 was cloned from the original PBMC derived virus provided by Francine McCutchan and Vicky Polonis (U.S. Military HIV Research Program). Env plasmids BaL.01 was recently described by our laboratory [26] and the Env used to generate the pseudovirus SS1196.1 was previously described [39]. The HIV-2 Env-pseudovirus 7312 containing the V343M mutation have previously been described [5]. The remaining functional Env plasmids were obtained from the NIH ARRRP.

### Human serum samples

Twenty randomly chosen serum samples were obtained via an MTA with the Global Solutions for Infectious Diseases. These sera were derived from volunteers from the VaxGen Inc phase III clinical trial. At the time of sampling (month 12.5) the participants had received four injections (month 0, 1, 6 and 12) with the AIDSVAX B/B vaccine containing 300 ug each of recombinant HIV-1_MN_ or HIV-1_GNE8_ derived gp120 in Alum adjuvant [13].

## Supporting Information

Figure S1 Biochemical analysis of the YU2 gp140-F trimers.
(3.17 MB TIF)

Figure S2 FACS gating strategy for cynomolgus macaque and human PBMCs.
(2.29 MB TIF)

Figure S3 HIV-2_7312/V434M_ (+9 nM sCD4) and HIV-1_MN_ neutralization by sera from monkeys immunized 2 times with gp140-F trimers formulated in the GSK Adjuvant System AS01B or in Ribi adjuvant.
(1.12 MB TIF)

Figure S4 V3 peptide or gp120 binding reactivity in serum samples from cynomolgus macaques, WT rabbits or huCD4 rabbits after immunization with gp140-F trimers.
(2.12 MB TIF)

Figure S5 Staining and gating strategy for flow cytometry analysis of rabbit PBMCs.
(2.12 MB TIF)
