# Supplementary figures and images for: B Cell Recognition of the Conserved HIV-1 Co-Receptor Binding Site Is Altered by Endogenous Primate CD4
Source: PLoS Pathog. 2008 Oct 3;4(10):e1000171. doi: 10.1371/journal.ppat.1000171 (PMC2542413; doi:10.1371/journal.ppat.1000171)

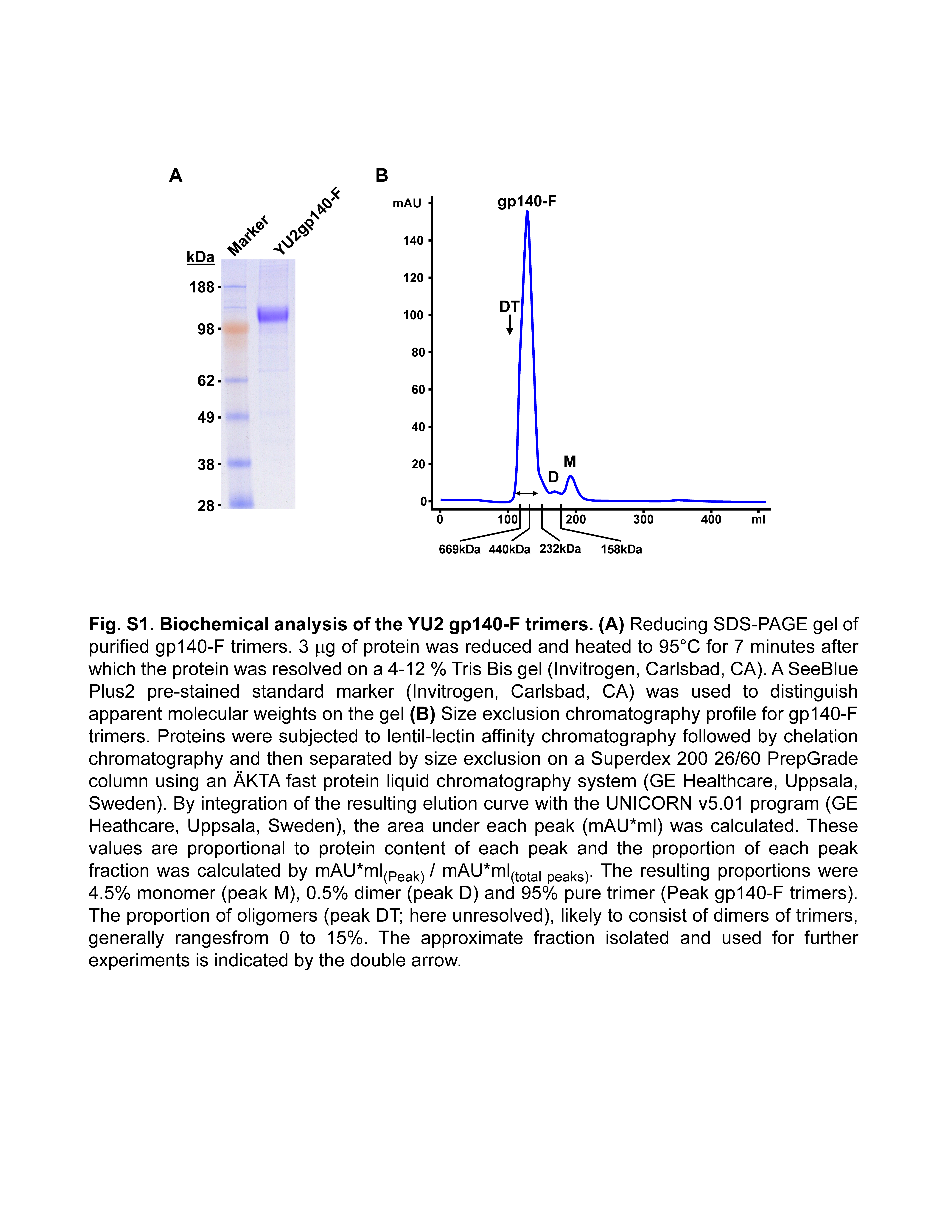

Supplement: Figure S1 — Biochemical analysis of the YU2 gp140-F trimers. (3.17 MB TIF) [file ppat.1000171.s001.tif]

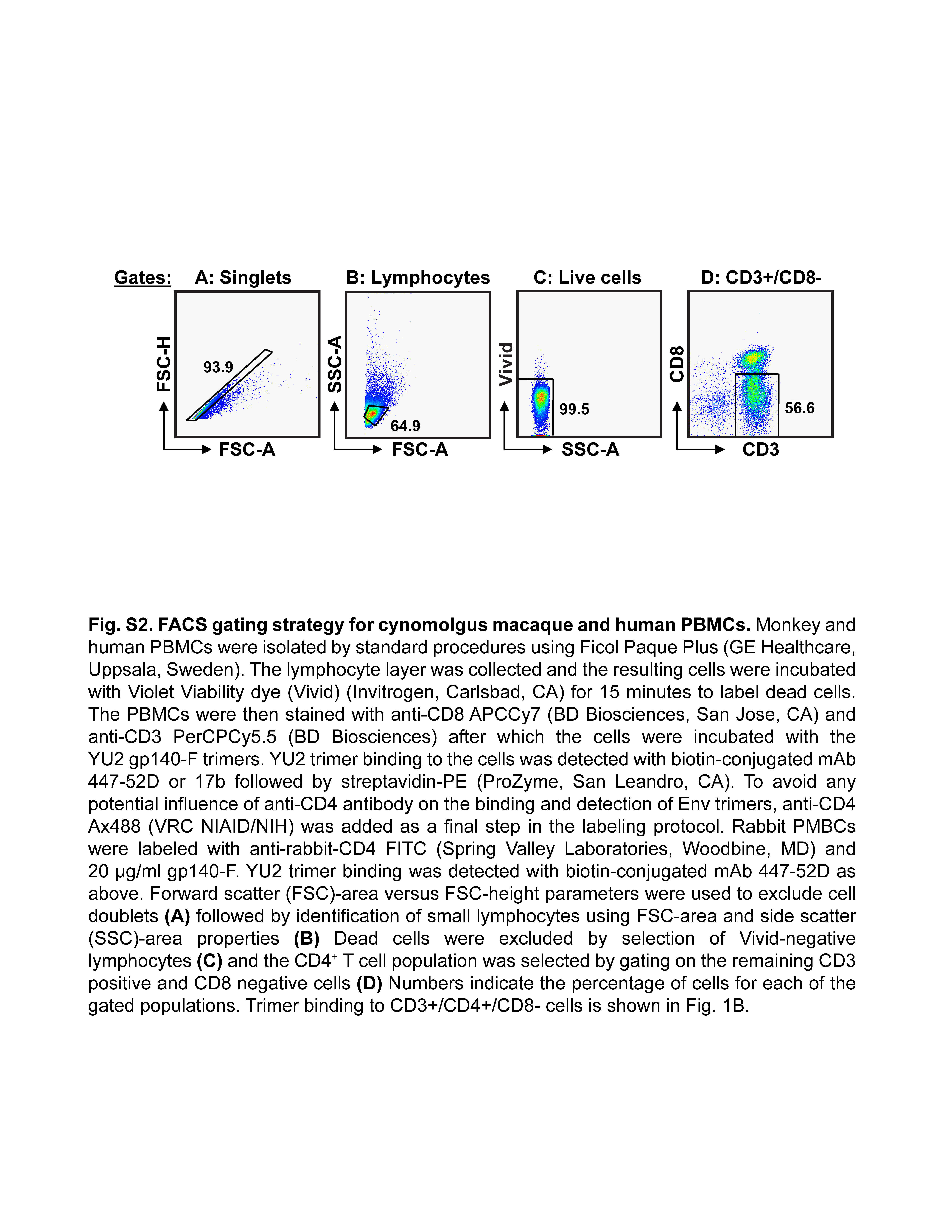

Supplement: Figure S2 — FACS gating strategy for cynomolgus macaque and human PBMCs. (2.29 MB TIF) [file ppat.1000171.s002.tif]

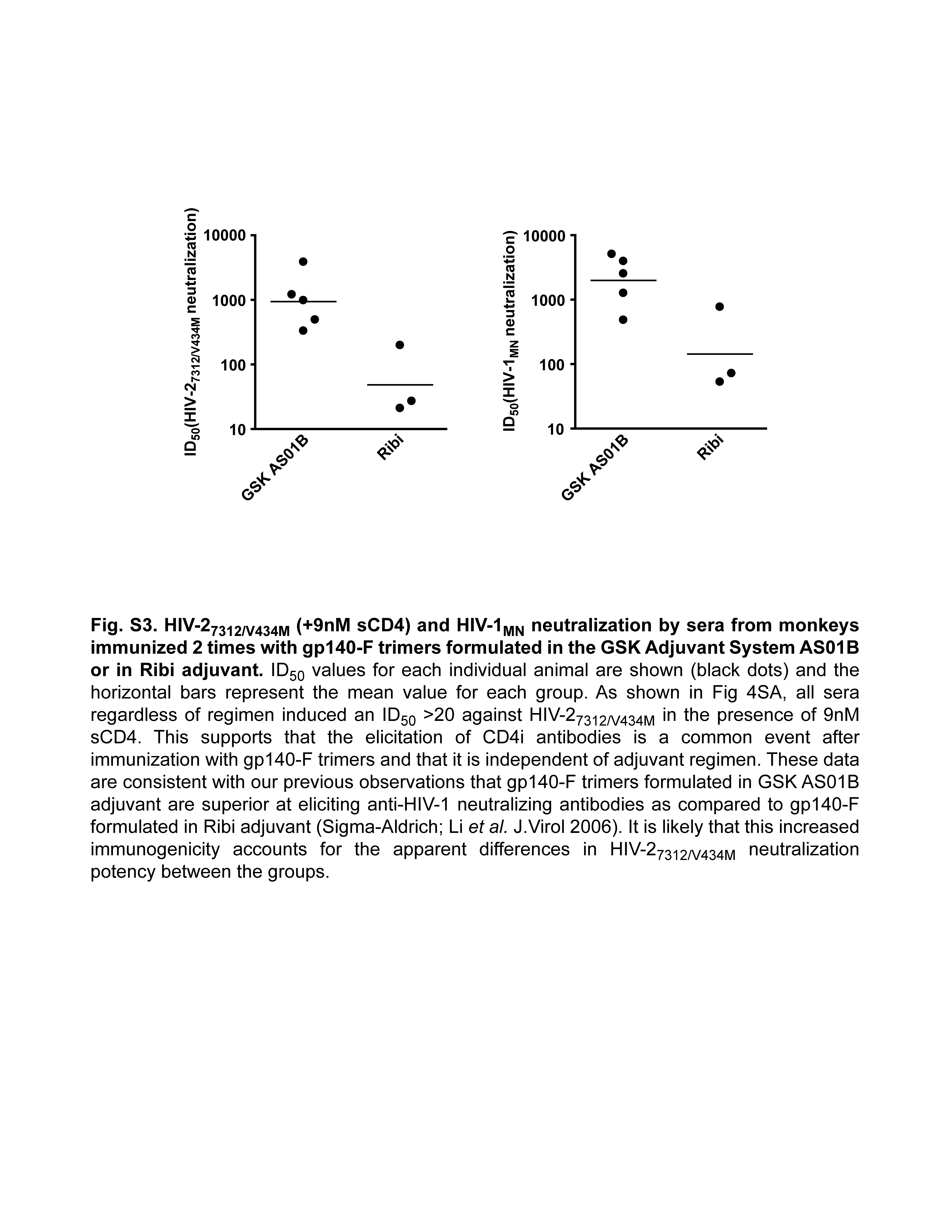

Supplement: Figure S3 — HIV-27312/V434M (+9 nM sCD4) and HIV-1MN neutralization by sera from monkeys immunized 2 times with gp140-F trimers formulated in the GSK Adjuvant System AS01B or in Ribi adjuvant. (1.12 MB TIF) [file ppat.1000171.s003.tif]

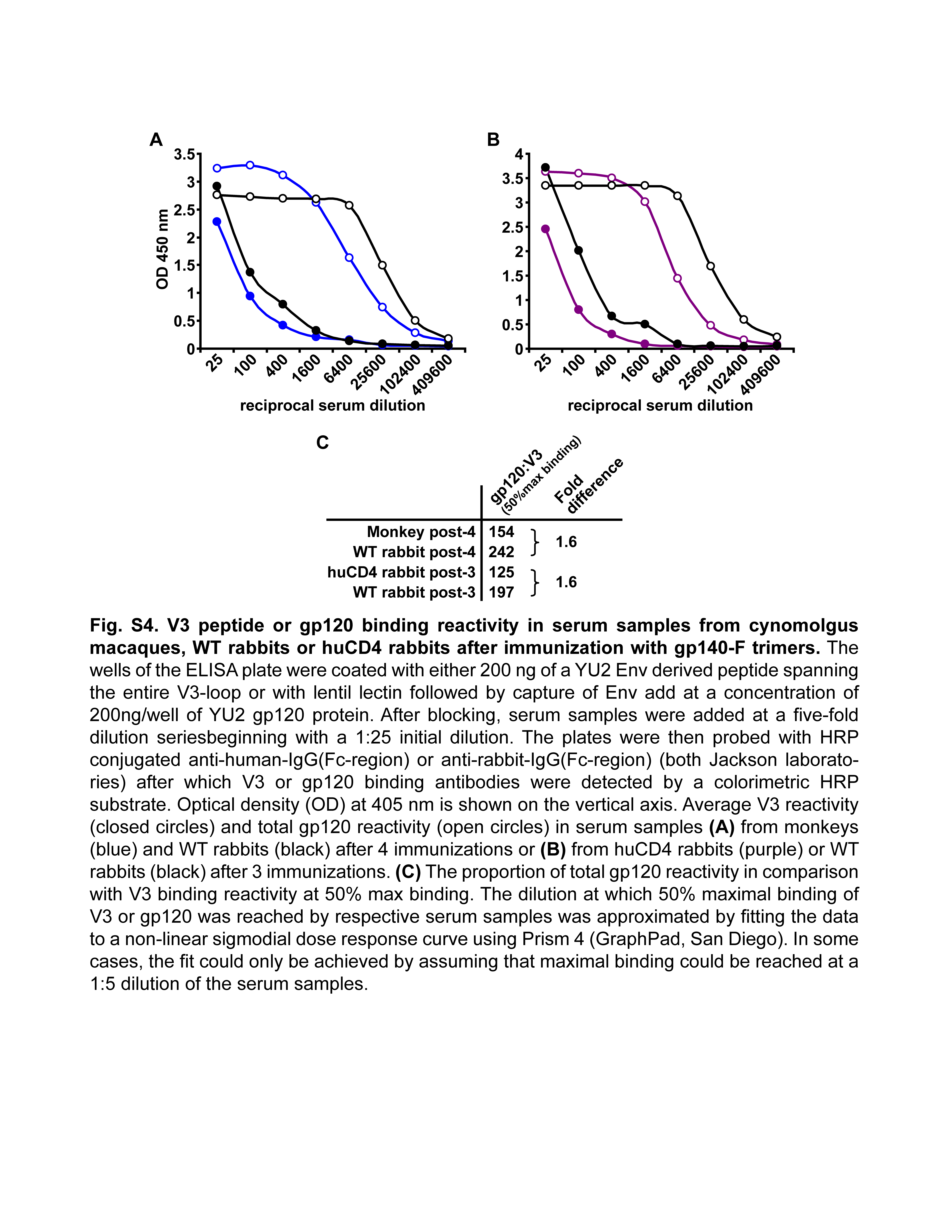

Supplement: Figure S4 — V3 peptide or gp120 binding reactivity in serum samples from cynomolgus macaques, WT rabbits or huCD4 rabbits after immunization with gp140-F trimers. (2.12 MB TIF) [file ppat.1000171.s004.tif]

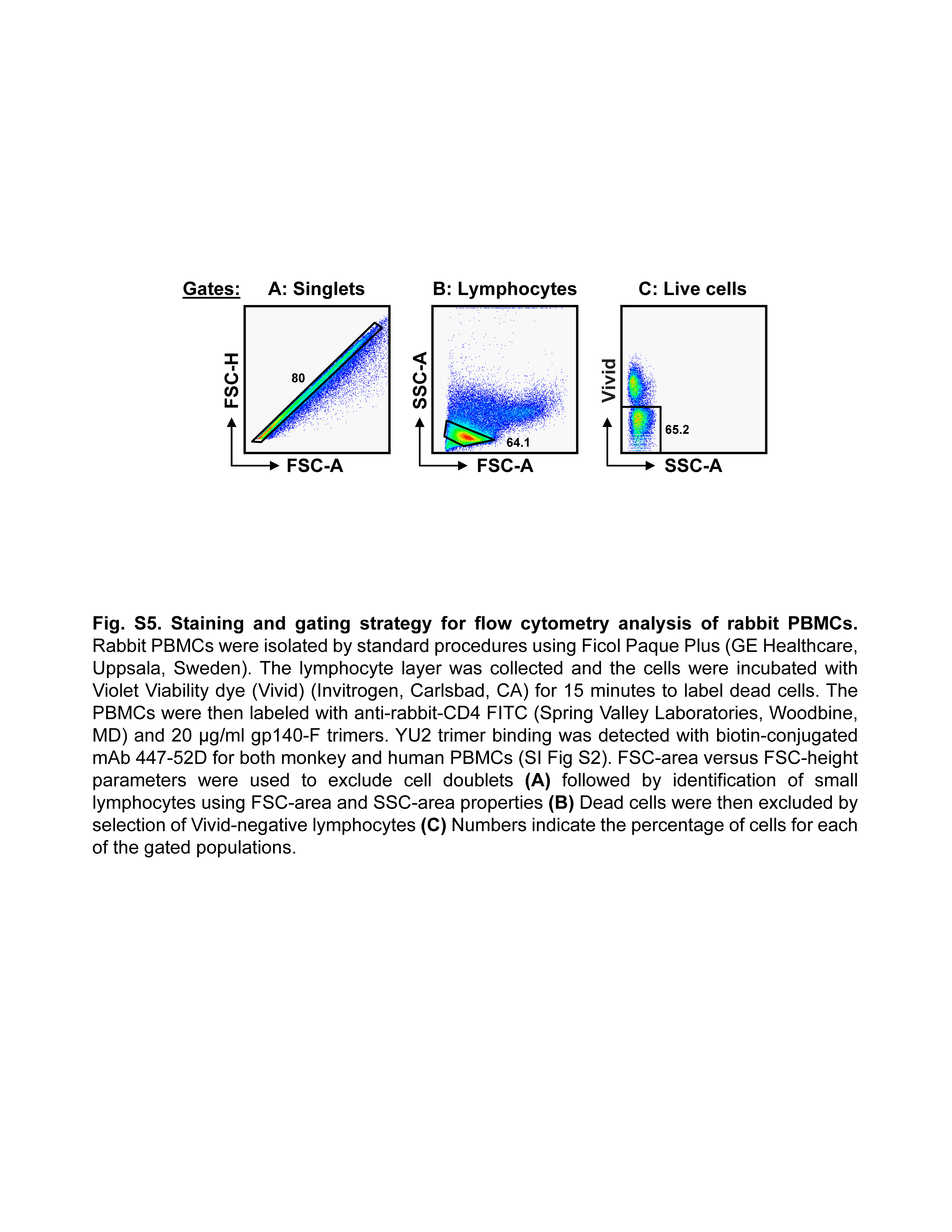

Supplement: Figure S5 — Staining and gating strategy for flow cytometry analysis of rabbit PBMCs. (2.12 MB TIF) [file ppat.1000171.s005.tif]
